# Evaluating the didactic value of 3D visualization in otosurgery

**DOI:** 10.1007/s00405-020-06171-9

**Published:** 2020-07-01

**Authors:** Nora M. Weiss, Armin Schneider, John M. Hempel, Florian C. Uecker, Sara M. van Bonn, Sebastian P. Schraven, Stefanie Rettschlag, Tobias Schuldt, Joachim Müller, Stefan K. Plontke, Robert Mlynski

**Affiliations:** 1 Department of Otorhinolaryngology, Head and Neck Surgery, “Otto Körner”, Rostock University Medical Center, Doberaner Strasse 137-139, 18057 Rostock, Germany; 2 ARRI Medical GmbH, Türkenstraße 89, 80799 Munich, Germany; 3 Research Group Minimally Invasive Interdisciplinary Therapeutical Intervention (MITI), “Klinikum rechts der Isar”, Technical University Munich (TUM), Munich, Germany; 4 Department of Otorhinolaryngology, Head and Neck Surgery, “Ludwig Maximilian University”, University Medical Center Munich, Munich, Germany; 5 Department of Otorhinolaryngology, Head and Neck Surgery, “Charité” University Medical Center, Berlin, Germany; 6 Department of Otorhinolaryngology, Head and Neck Surgery, Martin Luther University Halle-Wittenberg, Halle (Saale), Germany

**Keywords:** E-learning, 3D visualization, Medical education, 3D evaluation

## Abstract

**Introduction:**

Improvements of surgical visualization add value to the quality of clinical routine and offer the opportunity to improve surgical education of medical staff. The aim of this study was to determine whether otorhinolaryngology trainees gain additional comprehension of the anatomical structures and the surgical site when 3D visualization is used.

**Methods:**

Data were collected from ENT trainees of microsurgical courses of the middle ear, inner ear and lateral skull base at four university ENT departments (Charité (Berlin), Martin Luther University Halle-Wittenberg (Halle/Saale), Ludwig Maximilian University (Munich) and Rostock University Medical Center). Participants were asked to complete a questionnaire assessing the subjective value of identical surgical field visualization in 3D for surgeon and observer.

**Results:**

A total of 112 participants completed the questionnaire. The majority of participants stated a high additional value of 3D visualization compared to 2D visualization, with 75% fully agreeing to the statement that 3D visualization of the surgical field is superior to perceive the anatomical topography and structures compared to 2D representation. Participants encouraged the storage of data in online learning platforms.

**Conclusion:**

The results show that 3D visualization with identical imaging for surgeon and observer is a useful tool in teaching of microsurgery. It addresses perception of anatomical topography and structures as well as conception of the surgical workflow.

**Electronic supplementary material:**

The online version of this article (10.1007/s00405-020-06171-9) contains supplementary material, which is available to authorized users.

## Introduction

Electronic learning implements computer-assisted methods including digitally available educational material such as online courses or videos and is used increasingly in medical education [1, 2]. Teaching materials available in electronic format gained importance within the past years and offer the opportunity of easily accessible knowledge in an international setting. Their value needs to be evaluated under the aim of further improvement. Not only for students but also for physicians, international exchange of knowledge may improve the assessment of new methods and mutual assistance. Anatomical education is challenging for both, resident and teachers, especially in microscopic and fine structure surgery. Traditionally, anatomy and surgical steps are taught in theoretical lectures with or without augmentation by various media. In addition, students and residents observe common surgeries during their clinical period. Traditional lectures lack the opportunity of interactive participation. Interactive computer-based methods in combination with practical teaching have been shown to have a great potential in general anatomy education [3]. Recent reviews conclude that digital education tools are considered to be a useful instrument for medical education purpose and are not inferior to traditional lectures [4, 5]. Successful combination of both, digital and interactive tools and the knowledge of experienced teachers, is required to guarantee a good understanding of the anatomical structures and applying anatomical knowledge in clinical decisions [6]. Additionally, it has been found that 3D imaging facilitates the understanding of spatial information and anatomical orientation [7, 8]. 3D models gain importance in multiple disciplines to assist surgical training and anatomic comprehension [9–15]. Izard et al. concluded that 3D visualizing is a very helpful tool to learn surgical steps especially under the aspect of repetition [16]. The aim of this study was to determine whether participants of courses for microsurgery in otology gain additional comprehension of the anatomical structures and the surgical situs when a visualization is used which provides identical views for surgeon and observer in 3D. In this study, a fully digital surgical microscope was used to assess the additional value of 3D visualization for the understanding of otosurgery.

## Methods

Data were collected from participants attending microsurgical courses of the middle ear, inner ear and lateral skull base at four university ENT departments: Charité (Berlin), Martin Luther University Halle-Wittenberg (Halle/Saale), Ludwig Maximilian University (Munich) and Rostock University Medical Center, (Table 1). In each course, live surgeries of the middle ear, inner ear and lateral skull base were transmitted from the surgical theater to the lecture hall or from a senior surgeon’s microscope during the temporal bone courses. A digital surgical microscope (ARRISCOPE, ARRI Medical GmbH, Munich, Germany) with the capability to transmit a high-resolution 3D image was used. The image frame, size and surgical field overseen was identical for surgeon and observers. The live images were presented to the trainees on 65-inch 3D displays (LG 65EF9509, LG Electronics, Seoul, Republic of Korea). The displays were positioned in a way, that all participants had a direct view on the screens without any viewing angle distortions. All participants of the course observed the surgical procedures with passive (polarized) 3D glasses (Schleiter & Jauernig, Hamburg, Germany) in real-time audio-visual transmission. At the end of each course, participants were asked to complete a questionnaire assessing the subjective value of 3D visualization. The questionnaire included six questions concerning the comprehension of the anatomy and the surgical steps compared to 2D visualization, the additional value of surgical courses when 3D visualization was used, the subjective complaints and satisfaction observing surgery via 3D glasses and the usefulness/opportunity of storing surgical videos for of e-learning access (see supplementary material for original questionnaire). Answers were presented using a 5-point Rating scale (Likert scale) (full agreement = 5 points; agreement = 4 points; indecisive = 3 points; rather no agreement = 2 points; no agreement = 1 point).

**Table 1:** Participating departments and course type. Further, the number of participants completing the questionnaire at the individual courses is given

Date	Location	Course type	Participants
2020, January 17–18	University Medical Center “Charité”, Berlin	Live Surgery	5
2019, March 4–6	Martin Luther University Halle-Wittenberg (Halle/Saale)	Live Surgery	33
2017, March 20–23	Martin Luther University Halle-Wittenberg (Halle/Saale)	Live Surgery	11
2018, March 13–16	University Medical Center “Ludwig Maximilian University”, Munich	Temporal Bone Course	10
2017, April 01–06	University Medical Center “Ludwig Maximilian University”, Munich	Temporal Bone Course	10
2015, April 21–24	University Medical Center “Ludwig Maximilian University”, Munich	Temporal Bone Course	15
2018, March 12–14	University Medical Center “Otto Körner”, Rostock	Live Surgery	28

## Results

**Fig. 1:**
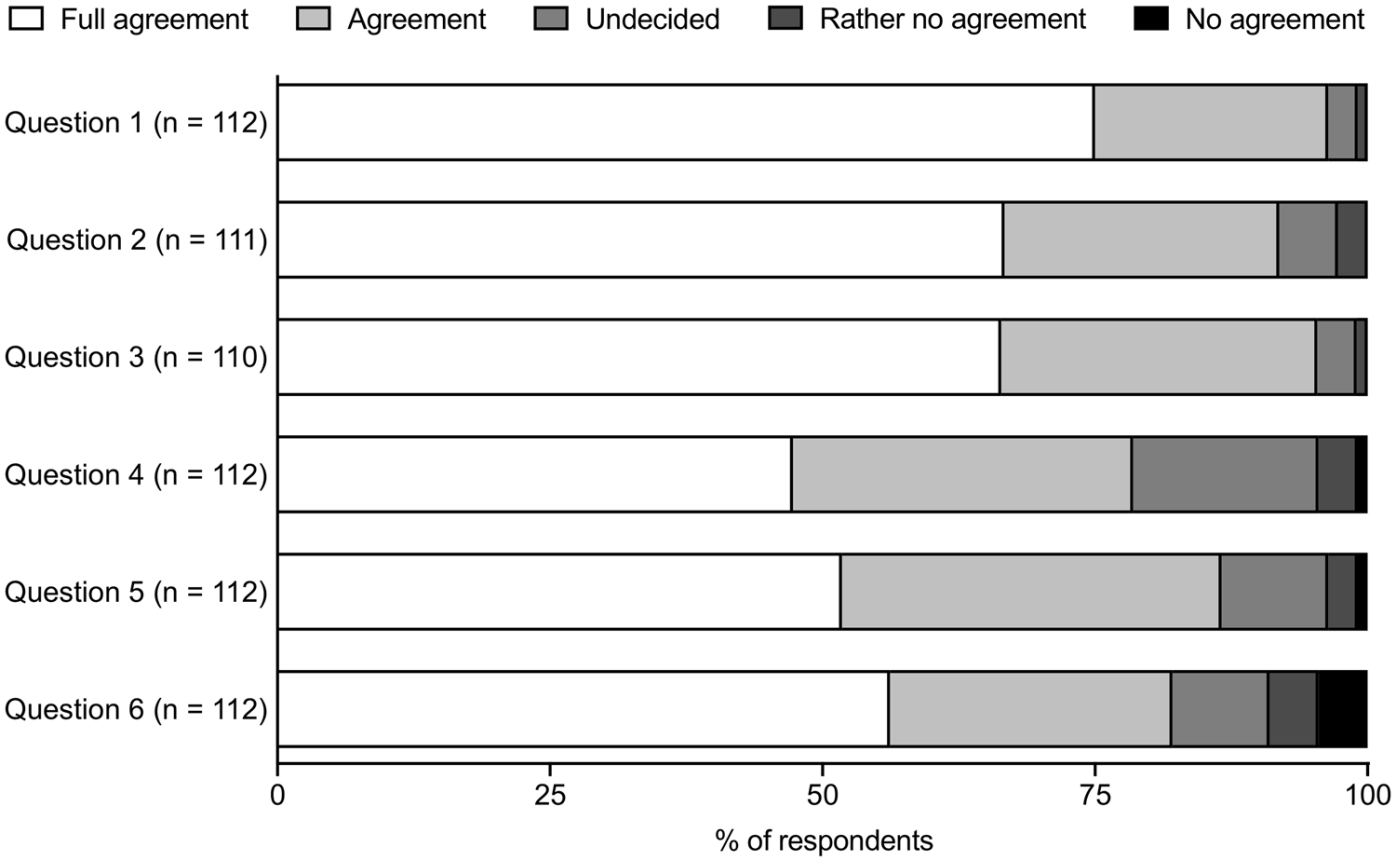
Overview of the participants’ answers to the individual questions (1–6). *N* indicates the number of participants answering the individual questions. Bars indicate the distribution of agreement from full agreement (white bar) to no agreement (black bar) in percent

**Fig. 2:**
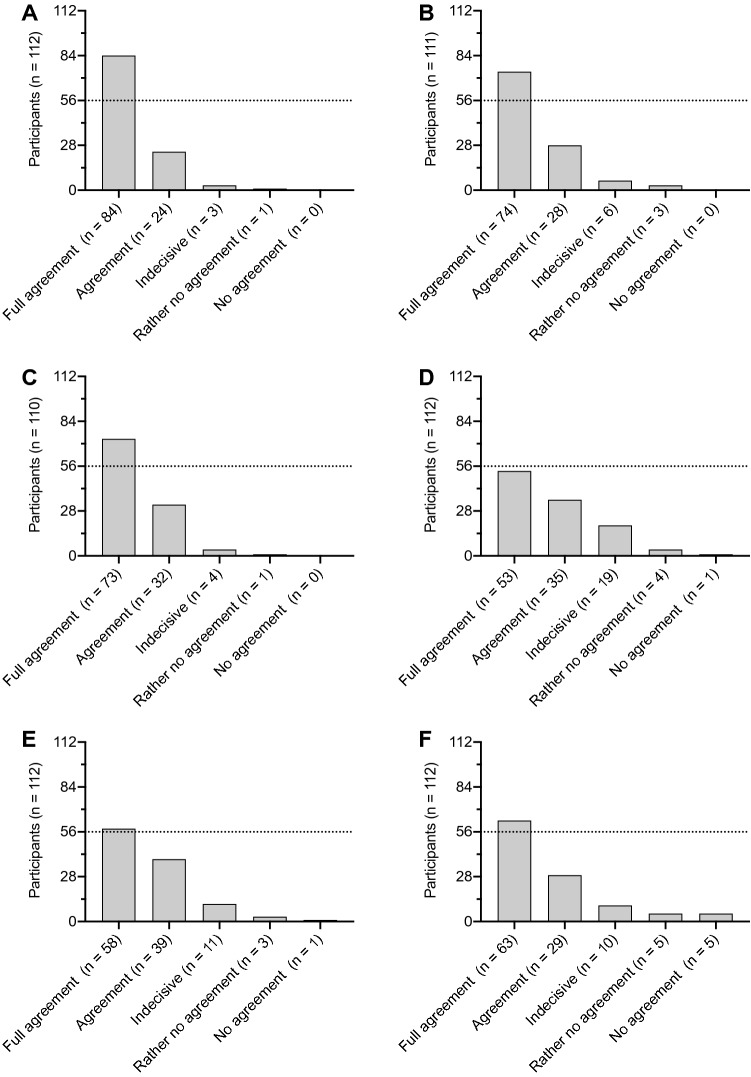
Answers of participants for individual statements (full agreement = 5 points; agreement = 4 points; indecisive = 3 points; rather no agreement = 2 points; no agreement = 1 point). N indicates the number of participants that answered the question. Dotted lines indicate 50% of participants. **a** Statement 1: “3D visualization of the surgical field was superior to perceive the anatomical topography and structures better compared to 2D representation.” **b** Statement 2: “3D visualization supported to follow the course and preparation of the surgical field compared to 2D representation.” **c** Statement 3: “The possibility to see the surgical field as a co-viewer in 3D provided additional value for surgical courses.” **d** Statement 4: “3D video data of a surgery should be archived for self-study and made available online for registered users.” **e** Statement 5 “The possibility to see the operation live via 3D video transmission at home would add value to online distance learning courses.” **f** Statement 6: “I had no problems such as indisposition, dizziness or headaches watching 3D films and videos.”

Data were available from seven microsurgical courses of the middle ear, inner ear and lateral skull base in four cities (Berlin, Halle, Munich, Rostock, all Germany) between 2015 and 2020. A total number of 112 participants completed the questionnaire. A total of 112 participants answered question 1, 4, 5 and 6, 111 participants answered question 2, and 110 participants answered question 3 (Table 2). Figure 1 shows an overview of the participants responses. Eighty-four participants (75%) fully agreed (5 points) to the statement that 3D visualization of the surgical field was superior to perceive the anatomical topography and structures better compared to 2D representation. Twenty-four participants (21.4%) agreed (4 points), 3 participants (2.7%) were indecisive (3 points), and 1 participant (0.9%) rather disagreed (2 points) to this statement (Fig. 2a).

**Table 2:** Descriptive statistics of the participants’ answers to the individual questions

Statement	1	2	3	4	5	6
Participants (*n*)	112	111	110	112	112	112
Minimum	2.0	2.0	2.0	1.0	1.0	1.0
25% percentile	4.3	4.0	4.0	4.0	4.0	4.0
Median	5.0	5.0	5.0	4.0	5.0	5.0
75% percentile	5.0	5.0	5.0	5.0	5.0	5.0
Maximum	5.0	5.0	5.0	5.0	5.0	5.0
Range	3.0	3.0	3.0	4.0	4.0	4.0
Mean	4.7	4.6	4.6	4.2	4.3	4.3
SD	0.05	0.07	0.06	0.09	0.08	0.10

Seventy-four participants (66.7%) fully agreed (5 points) to the statement that 3D visualization supported to follow the course and preparation of the surgical field compared to 2D representation. Twenty-eight participants (25.2%) agreed (4 points), 6 participants (5.4%) were indecisive (3 points), and 3 participants (2.7%) rather disagreed (2 points) to this statement (Fig. 2b).

Seventy-three participants (66.4%) fully agreed (5 points) to the statement that the possibility to see the surgical field as a co-viewer in 3D provided additional value for surgical courses. Thirty-two participants (29%) agreed (4 points), 4 participants (3.6%) were indecisive (3 points), and 1 participant (1%) rather disagreed (2 points) to this statement (Fig. 2c).

Fifty-three participants (47.3%) fully agreed (5 points) to the statement that the possibility to see the operation live via 3D video transmission at home would add value to online distance learning courses. Thirty-five participants (31.2%) agreed (4 points), 19 participants (17.0%) were indecisive (3 points), 4 participants (3.6%) rather disagreed (2 points), and 1 participant (0.9%) disagreed (1 points) to this statement (Fig. 2d).

Fifty-eight participants (51.8%) fully agreed (5 points) to the statement that 3D video data of a surgery should be archived for self-study and made available online for registered users. Thirty-nine participants (34.8%) agreed (4 points), 11 participants (9.8%) were indecisive (3 points), 3 participants (2.7%) rather disagreed (2 points), and 1 participant (0.9%) disagreed (1 points) to this statement (Fig. 2e).

Sixty-three participants (56.2%) fully agreed (5 points) to the statement that they had no problems such as indisposition, dizziness or headaches watching 3D films and videos. Twenty-nine participants (25.9%) agreed (4 points), 10 participants (8.9%) were indecisive (3 points), 5 participants (4.5%) rather disagreed (2 points), and 5 participants (4.5%) disagreed (1 points) to this statement (Fig. 2f).

## Discussion

Today, the majority of microsurgical procedures are carried out using conventional optical surgical microscopes allowing a stereoscopic view using binocular vision through the microscope. The disparity of the eyes generates two retinal pictures that are recombined into a 3D vision by the brain. Most microscopes are mounted with a beam splitter providing an additional monocular view for trainees or surgical assistants. In some cases, they are mounted to a camera that enables other observers to follow surgery on a separate screen. Nevertheless, it is known, that, even in subjects that miss the ability of stereovision, binocular vision is advantageous when learning surgical skills [17]. Improvements of surgical visualization not only add value to the quality of clinical routine but also offer the opportunity to improve the surgical education of medical staff. These findings were encouraged by the results of this study where the majority of participants rated an overall improvement in their comprehension of the course content when providing the opportunity of 3D-visualization. Evaluating the students’ subjective perceptions of learning is regarded as an useful indicator of teaching success and becomes part of educational resource development [18, 19]. Particularly in microsurgery, precise work and detailed anatomical knowledge is mandatory. Tools for an efficient and safe training of the surgical steps and the microsurgical environment are needed. The aim during training and teaching is to improve the comprehension of anatomical details, distances and orientation between microstructures within the surgical field and the individual surgical proportions of the surgical site together with the sequence of surgical steps for the observer. As a result of this study, it is worthwhile to transfer an image equal to the surgeon’s view onto large displays that feature 3D visualization for teaching purposes. This enables trainees to follow the surgery with a view identical to that of the surgeon. It has been shown that stereo vision improves the ability to perform tasks using a surgical microscope or surgery simulator [19]. By providing additional textural information about the anatomical structures, an improvement of knowledge about morphological and pathological information is assumed and offers the option of repetitional learning [16]. Technical advancements offer assistance to help students, residents or fellows develop new skills. Fully digital microscopes bear multiple opportunities to add value to surgical routine and assist the visualization for other observers.

E-learning has gained importance over the past years, especially under the aim of internationally uniform standards [20, 21]. Modern concepts that implement digitization into the curricular ENT education of students show an overall high interest and positive acceptance of the topic [22]. Nevertheless, the format of e-learning requires a good conceptualization in order not to exceed a critical information content [23]. In this study, broad and comprehensible conceptualization was approached by the generation of high-resolution video material and its displaying on 65-inch screens led to high user satisfaction is this study. A recent survey reported high demands on surgical instruction videos [18]. This task is easily achievable when using the record function of the digital microscope. Surgery may be recorded and uploaded to educational databases. However, the importance of high-quality teaching material needs to be considered. The present study addresses both, the additional value of 3D visualization and the availability of e-learning material. Seventy-five percent of the participants fully agreed to the superiority of 3D compared to 2D visualization concerning the perception of anatomical topography and structures. Additionally, 3D visualization was rated to support the ability to follow the course and preparation. Furthermore, the participants encouraged the availability of educational databases. These findings are in accordance with the recent literature addressing the applicability and evaluation of additional modern education instruments for students and residents that aim to improve their performance in practice [24–26]. Using more realistic assessments may add value to existing teaching methods. For future structuring of surgical education, the advantages of 3D visualization should be utilized. Potential disadvantages such as the costs of the equipment and the requirements for data transfer or storage need to be opposed to the value of safe and fast comprehension of anatomy and procedures.

This study has several limitations: First, the participants were not tested for their ability of stereo vision. Nevertheless, it can be assumed that the high questionnaire scores and the low scores for discomfort when using 3D glasses indicate that at least the majority of participants owned the ability of stereo vision. Furthermore, a control group having 2D vision only was not tested. Since data were obtained from educational courses, the high standards did not allow a study design with a control group. For this reason, the questionnaire was designed to ask questions about the subjective benefit from 3D visualization only. Nonetheless, for a substantial part of the participants 2D monitors were available at the individual practice sites giving the opportunity to discuss individual findings while preparing the temporal bone specimen. Thus, a direct comparison of 2D- and 3D visualization was principally given. Further studies on the effectiveness of 3D visualization for teaching should use protocols with randomized study groups for 2D and 3D visualization. The groups could be tested for prior knowledge. A catalog of learning objectives and target parameters should be defined.

## Conclusions

The results show that 3D imaging with identical view for surgeon and observer is a useful tool in microscopical surgery education in terms of perceiving the anatomical topography and structures and in understanding the surgical workflow. 3D visualization and e-learning access of surgical videos need to be encouraged.

## Electronic supplementary material

Below is the link to the electronic supplementary material.

Supplementary material 1 (TIFF 33976 kb)

## References

[1] Sandars J, Walsh K, Sandars J, Lakhani M, Banks I (2006). E-learning for general practitioners: lessons from the recent literature. *E*-*Learning for GP educators*.

[2] Sieber D, Erfurt P, John S (2019). The OpenEar library of 3D models of the human temporal bone based on computed tomography and micro-slicing. Sci Data..

[3] Stanford W, Erkonen WE, Cassell MD (1994). Evaluation of a computer-based program for teaching cardiac anatomy. Invest Radiol.

[4] Cook DA, Levinson AJ, Garside S, Dupras DM, Erwin PJ, Montori VM (2008). Internet-based learning in the health professions: a meta-analysis. JAMA.

[5] Kyaw BM, Posadzki P, Dunleavy G (2019). Offline digital education for medical students: systematic review and meta-analysis by the digital health education collaboration. J Med Internet Res..

[6] de Leng BA, Dolmans DHJM, Muijtjens AMM, van der Vleuten CPM (2006). Student perceptions of a virtual learning environment for a problem-based learning undergraduate medical curriculum. Med Educ.

[7] Chhaya N, Helmy O, Piri N, Palacio A, Schaal S (2018). Comparison of 2D and 3D video displays for teaching vitroretinal surgery. Retina..

[8] de Boer IR, Wesselink PR, Vervoorn JM (2016). Student performance and appreciation using 3D vs 2D vision in a virtual learning environment. Eur J Dent Educ..

[9] Chou P-Y, Hallac RR, Shih E (2018). 3D-Printed Models of Cleft Lip and Palate for Surgical Training and Patient Education. Cleft Palate Craniofac J.

[10] Kleinert R, Wahba R, Chang D-H, Plum P, Holscher AH, Stippel DL (2015). 3D immersive patient simulators and their impact on learning success: a thematic review. J Med Internet Res..

[11] Barber SR, Kozin ED, Naunheim MR, Sethi R, Remenschneider AK, Deschler DG (2018). 3D-printed tracheoesophageal puncture and prosthesis placement simulator. Am J Otolaryngol.

[12] Messier E, Wilcox J, Dawson-Elli A, Diaz G, Linte CA (2016). An interactive 3D virtual anatomy puzzle for learning and simulation—initial demonstration and evaluation. Stud Health Technol Inform..

[13] Reymus M, Fotiadou C, Kessler A, Heck K, Hickel R, Diegritz C (2019). 3D printed replicas for endodontic education. Int Endod J.

[14] Wang J-L, Yuan Z-G, Qian G-L, Bao W-Q, Jin G-L (2018). 3D printing of intracranial aneurysm based on intracranial digital subtraction angiography and its clinical application. Medicine (Baltimore)..

[15] Roosli C, Sim JH, Mockel H, Mokosch M, Probst R (2013). An artificial temporal bone as a training tool for cochlear implantation. Otol Neurotol.

[16] Izard SG, Juanes JA, Garcia Penalvo FJ, Estella JMG, Sanchez Ledesma MJ, Ruisoto P (2018). Virtual reality as an educational and training tool for medicine. J Med Syst.

[17] van Mierlo CM, Brenner E, Smeets JBJ (2011). Better performance with two eyes than with one in stereo-blind subjects’ judgments of motion in depth. Vision Res.

[18] Shabli S, Heuermann K, Leffers D (2019). Survey on the need for an e-learning-platform for ENT residents. Laryngorhinootologie.

[19] Nibourg LM, Wanders W, Cornelissen FW, Koopmans SA (2015). Influence of stereoscopic vision on task performance with an operating microscope. J Cataract Refract Surg.

[20] Lau F, Bates J (2004). A review of e-learning practices for undergraduate medical education. J Med Syst.

[21] Chumley-Jones HS, Dobbie A, Alford CL (2002). Web-based learning: sound educational method or hype? A review of the evaluation literature. Acad Med.

[22] Offergeld C, Neudert M, Emerich M, Schmidt T, Kuhn S, Giesler M (2020). Mediation of data literacy in curricular education in otorhinolaryngology: watch and wait or anticipatory obedience?. HNO..

[23] Daubenfeld T, Kromeier J, Heermann S, Hildenbrand T, Giesler M, Offergeld C (2020). Traditional vs. modern: possibilities and limitations of the new lecture concept in ENT teaching curricula. HNO..

[24] Wijnen-Meijer M, Gartmeier M, Berberat PO (2020). Overview on research in the field of medical education. HNO..

[25] Gaupp R, Fabry G, Korner M (2018). Self-regulated learning and critical reflection in an e-learning on patient safety for third-year medical students. Int J Med Educ..

[26] Gaupp R, Korner M, Fabry G (2016). Effects of a case-based interactive e-learning course on knowledge and attitudes about patient safety: a quasi-experimental study with third-year medical students. BMC Med Educ.

