# Supplementary figures and images for: Evaluating the didactic value of 3D visualization in otosurgery
Source: Eur Arch Otorhinolaryngol. 2020 Jul 1;278(4):1027–33. doi: 10.1007/s00405-020-06171-9 (PMC7954761; doi:10.1007/s00405-020-06171-9)

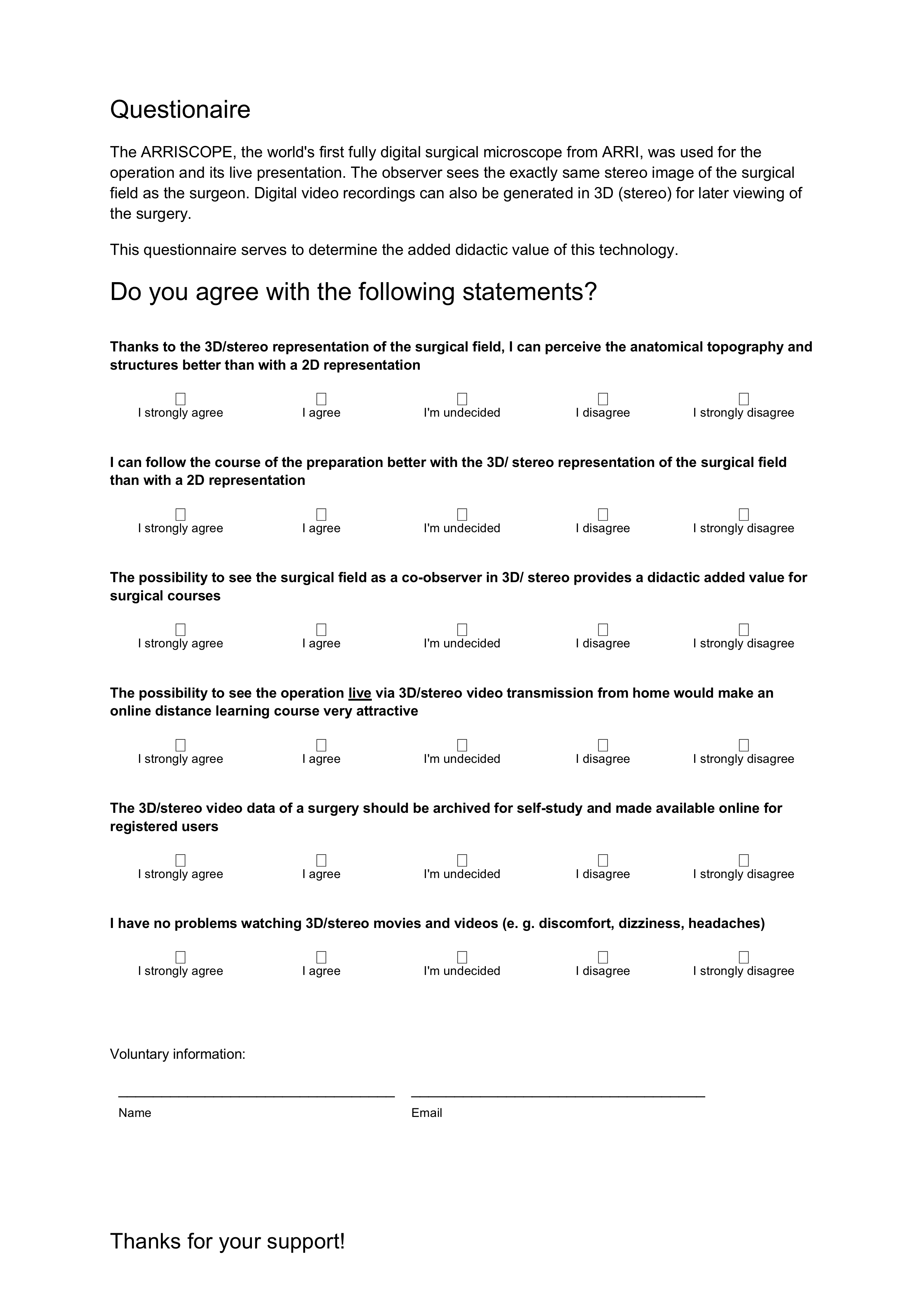

Supplement: Supplementary file 1 — Supplementary material 1 (TIFF 33976 kb) [file 405_2020_6171_MOESM1_ESM.tiff]
